# Machine Learning‐Based Classification of Right and Left Ventricular Outflow Tract Premature Ventricular Contractions

**DOI:** 10.1111/anec.70250

**Published:** 2026-10-26

**Authors:** Michail Botis, Dimitris Tsiachris, George G. Botis, Aristomenis Koumpanakis, Lamprini Iro Bartsioka, Ioannis Doundoulakis, Athanasios Kordalis, Christos‐Konstantinos Antoniou, Aikaterini‐Eleftheria Karanikola, George K. Matsopoulos, Konstantinos Tsioufis

**Affiliations:** ^1^ First Department of Cardiology “Hippokration” Hospital, National and Kapodistrian University Athens Greece; ^2^ Athens Heart Center Athens Medical Center Athens Greece; ^3^ Biomedical Engineering Laboratory, School of Electrical and Computer Engineering National Technical University of Athens Zografou Greece

**Keywords:** catheter ablation, ECG classification, machine learning, ventricular arrhythmias

## Abstract

**Introduction:**

Idiopathic premature ventricular contractions (PVCs) commonly originate from the right and left ventricular outflow tracts (RVOT and LVOT). The surface 12‐lead electrocardiogram (ECG) is commonly used to differentiate the anatomic site of origin prior to catheter ablation. Machine learning methods can further enhance diagnostic accuracy.

**Materials and Methods:**

In this study, 64 ECG‐derived features, along with age and gender, were analyzed from 43 patients (65% males, median age 55.6, IQR 30 years), who underwent successful catheter ablation of outflow tract‐originating idiopathic PVCs. The site of origin was depicted through 3‐D electroanatomical mapping. A logistic regression model was trained to classify the site of origin as either RVOT or LVOT using repeated stratified five‐fold cross‐validation with five repeats. A model restricted to ECG features and four previously published ECG algorithms was evaluated for comparison.

**Results:**

Across the 25 held‐out partitions, the model achieved an accuracy of 0.817 ± 0.143, precision of 0.888 ± 0.122, recall of 0.824 ± 0.206, F1‐score of 0.836 ± 0.139 and ROC‐AUC of 0.917 ± 0.094. Using the pooled out‐of‐fold predictions, accuracy was 0.860 (95% CI 0.744–0.953) and ROC‐AUC 0.910 (95% CI 0.800–0.987). Age was the most discriminative feature, with increasing values favoring LVOT origin. The ECG‐only model achieved an ROC‐AUC of 0.853 (95% CI 0.721–0.958; ΔAUC +0.057, *p* = 0.104).

**Conclusions:**

These findings suggest that machine learning models, based on ECG parameters and demographic characteristics, can classify outflow tract ventricular arrhythmias, supporting their use in automated differentiation and pre‐procedural planning.

## Graphical abstract

Machine‐learning analysis of ECG‐derived features and demographic variables enables accurate and interpretable differentiation between RVOT‐ and LVOT‐originating premature ventricular contractions.

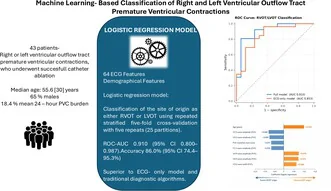

## 1 Introduction

Idiopathic premature ventricular contractions (PVCs) are one of the most frequently encountered clinical arrhythmias. Two common sites of origin are the right and left ventricular outflow tracts (RVOT and LVOT, respectively) (Tsiachris et al. 2023). Radiofrequency catheter ablation (RFCA) is an effective therapeutic modality in symptomatic patients or a worsened cardiac function attributed to frequent PVCs (Zeppenfeld et al. 2022). Accurate preprocedural localization is important in terms of procedural efficiency and lowering of procedural risks (Cronin et al. 2019). However, anatomically juxtaposed RVOT and LVOT sites may produce highly similar ECG patterns, rendering the ECG localization of outflow tract PVCs challenging (Anderson et al. 2019).

Traditionally, electrocardiogram (ECG)‐derived rules and algorithms have been developed to predict LVOT vs. RVOT origin of outflow tract PVCs. Some of the most commonly cited include the R/S–wave amplitude index (Zhao et al. 2022), the transitional zone index (Yoshida et al. 2011), the V2S/V3R index (Yoshida et al. 2014) and the V2 transition ratio (Betensky et al. 2011). None of these algorithms has achieved perfect discrimination, and challenges regarding practical and reproducible implementation are posed.

In recent years, the application of artificial intelligence (AI) in cardiology has evolved dramatically. Machine Learning (ML) models have been applied to various aspects. In the arrhythmias field, models that can simultaneously evaluate data from multiple leads to predict PVC origin have been proposed (Nakasone et al. 2024; Wang et al. 2024). However, ECG features previously defined as distinguishing between RVOT‐ and LVOT‐originating PVCs have not yet been systematically integrated into machine‐learning models. Moreover, the interpretability of ML models is a critical consideration in clinical practice, where understanding the rationale behind a prediction is essential for decision‐making.

The aim of this study was to integrate electrocardiographic features characteristic of RVOT‐ and LVOT‐originating PVCs, as defined by previously published conventional algorithms, together with demographic parameters previously shown to discriminate between RVOT and LVOT origins—specifically age and sex (Botis et al. 2025; Penela et al. 2023)—into a novel machine‐learning model. To this end, a logistic regression model was developed and trained, using a curated set of ECG‐derived features. In addition, feature‐importance analysis was performed to identify the most influential predictors of PVC origin.

## 2 Materials and Methods

### 2.1 Study Population

The study cohort included patients with documented premature ventricular contractions, originating from either RVOT or LVOT, who underwent catheter ablation, from June 2019 to September 2024, at two tertiary healthcare centers in Greece. Preoperatively, 12‐lead ECG data, along with demographic characteristics and comorbidities, were collected for all the patients. Patients with ECG morphologies other than outflow tract originating PVCs were excluded.

### 2.2 Mapping and Ablation Protocol

The ablation procedure was performed without sedation. Electroanatomic mapping system [CARTO3 (Biosense Webster, Diamond Bar, CA, USA)] was used. After written informed consent, a 6‐F quadripolar catheter was inserted via the femoral vein and placed through the atrioventricular valve to map the largest His potential. Pace and activation mapping were performed using either a 7‐French, 4‐mm–tip non‐irrigated ablation catheter or a 7.5‐French, 3.5‐mm–tip irrigated ablation catheter. Mapping was initially conducted in the right ventricle, and in cases where favorable mapping characteristics were not identified, or ablation failed to abolish the arrhythmia, mapping was subsequently extended to the left ventricle, through a retrograde aortic approach. When few PVCs were observed at the beginning of the electrophysiologic study, induction was attempted with isoproterenol infusion. Activation mapping was performed in all patients during the PVCs. Pace mapping was performed at the maximum output (1.0 ms, 20 V), and the output was decreased until loss of capture. The ablation site was determined using a combination of pace mapping (> 11/12 leads), electrogram precedence of the earliest bipolar ventricular electrogram relative to the QRS onset and the initial QS morphology for unipolar ventricular electrogram during PVCs. The radiofrequency current was delivered with an ablation catheter, with a power setting of 25–40 W and a temperature limit of 43°C. Contact force‐sensing technology was used, with a target of 5–30 g contact force. If the PVC disappeared or the frequency of the arrhythmias diminished after the first 30 s of ablation, the energy was delivered continuously for 60 s. Procedural success was defined as the absence of spontaneous or induced PVCs at 30 min after the last energy delivery, and was confirmed by continuous cardiac monitoring over the subsequent 24 h of inpatient care. The origin of OT‐VAs was determined based on the 3D electroanatomical mapping and the response to ablation. Systemic anticoagulation was achieved with intravenous heparin targeting a minimum activation clotting time of 350 s.

### 2.3 Data Collection and Preprocessing

Electrocardiogram ECG features, previously incorporated on traditional ECG algorithms differentiating LVOT and RVOT originating arrhythmias (Zhao et al. 2022; Yoshida et al. 2011, 2014; Betensky et al. 2011; Cheng et al. 2013; Di et al. 2019; He et al. 2018; Kaypakli et al. 2018; Nikoo et al. 2020), were manually extracted by a certified cardiologist, blinded to arrhythmia origin. The following measures were obtained for both SR and PVC: (1) R‐ and S‐wave amplitudes in leads I, II, III, aVF, aVL, aVR and V1 to V6; (2) R‐wave and S‐ wave duration in leads V1 to V3; (3) QRS duration; and (4) QRS inferior or superior axis. In addition to ECG features, demographic data including age and gender were incorporated into the analysis. All data processing, model training, and evaluation were performed using Python libraries: NumPy (version 1.26.4), pandas (version 2.2.1), SciPy (version 1.12.0), and scikit‐learn (version 1.4.1).

### 2.4 Development of the ML Model

Model training was performed using a nested five‐fold cross‐validation scheme, repeated five times with different random partitions and stratified on outflow tract origin, yielding 25 held‐out partitions. All data‐dependent preprocessing, including z‐score normalization and feature selection, was performed within each training partition only, excluding the corresponding held‐out fold. Feature selection used the ANOVA F‐test, with each candidate feature scored according to its F‐statistic; the eight features with the highest F‐scores were retained to maintain a robust feature‐to‐sample ratio and mitigate the risk of overfitting. Within the same partitioning scheme, logistic regression hyperparameters—regularization strength (*C* = 0.01, 0.1, 1.0, 10.0, 100.0) and penalty type (L1, L2), using the liblinear solver—were optimized by inner cross‐validation, with the area under the receiver operating characteristic curve (AUC) as the objective. The model was also refitted to the complete cohort, with hyperparameters selected by cross‐validation within that cohort, in order to obtain model coefficients for interpretation (Figure 1).

**FIGURE 1:**
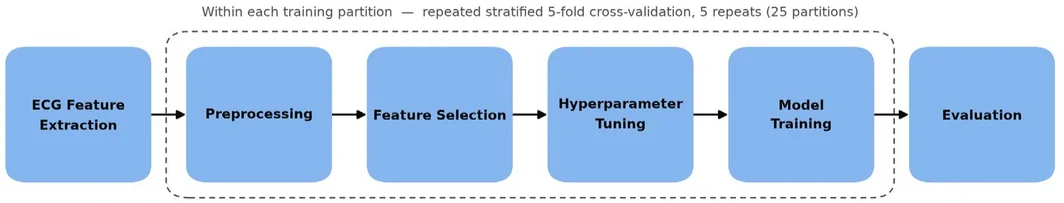
Overview of the study workflow.

Accuracy, precision, recall, F1‐score, and AUC were computed in each of the 25 held‐out partitions and are reported as mean ± standard deviation. Ninety‐five percent confidence intervals for AUC and accuracy were estimated by bootstrap resampling (2000 iterations, with replacement).

### 2.5 Model Comparison

Two models were developed. The reference model included ECG features, age, and sex. The ECG‐only model included ECG features alone. The reference model was additionally compared with four previously published electrocardiographic algorithms: the R/S‐wave amplitude index (Zhao et al. 2022), the transitional zone index (Yoshida et al. 2011), the V2S/V3R index (Yoshida et al. 2014) and the V2 transition ratio (Betensky et al. 2011). Each was applied at its originally published cut‐off; as these algorithms involve no fitting, they were evaluated directly in the whole cohort.

### 2.6 Model Evaluation

Accuracy, precision, recall, F1‐score and AUC were computed in each of the 25 held‐out partitions and are reported as mean ± standard deviation. In addition, the five out‐of‐fold predicted probabilities obtained for each patient were averaged, and these patient‐level predictions were used to compute the same metrics together with the confusion matrix, applying a probability threshold of 0.5. Ninety‐five percent confidence intervals for the patient‐level estimates were obtained by bootstrap resampling of patients (2000 iterations, with replacement).

Differences in AUC between the two models and between the full model and each published algorithm were assessed using DeLong's test for correlated ROC curves.

## 3 Results

### 3.1 Patient Characteristics and Procedural Data

The study cohort included 43 patients with documented outflow tract ventricular arrhythmias, comprising 26 (60.5%) patients with LVOT‐originating arrhythmia and 17 (39.5%) patients with RVOT‐originating arrhythmia. The baseline patient characteristics are summarized in Table 1. The median age was 55.6 (IQR 30) years, and 28 (65.1%) patients were males. The median left ventricular ejection fraction (EF) was 55 (IQR 10).

**TABLE 1:** Patient baseline characteristics.

	All (*n* = 43)	RVOT (*n* = 17)	LVOT (*n* = 26)	*p*
Age (years)	55.6 (30)	41.2 (16.1)	66.1 (9.6)	< 0.001
Gender (male)	28 (65%)	11 (65%)	17 (65%)	> 0.9
LVEF	55 (10)	60 (5)	55 (11)	0.07
PVC 24‐h burden (%)	18.5 (14.4)	17 (30)	19 (12.4)	> 0.9
Structural heart disease	9 (21%)	2 (12%)	7 (28%)	0.3
Procedural duration (min)	120.0 (40.0)	120.0 (70.0)	120.0 (60.0)	> 0.9
Fluoroscopy duration (min)	2.0 (3)	1.0 (4.5)	2.0 (3.0)	0.5
Failure of beta‐blocker therapy	16 (37.2%)	6 (35.3%)	10 (38.5%)	> 0.9
Failure of Class Ic—antiarrhythmic drugs therapy	12 (27.9%)	6 (35.3%)	6 (23.1%)	0.49
Failure of Class III—antiarrhythmic drugs therapy	3 (7.0%)	0	3 (11.5%)	0.27

*Note:* Data are presented as the median (IQR) or *n* (%).

Abbreviations: LVEF, left ventricular ejection fraction; PVC, premature ventricular contraction.

### 3.2 Feature Selection and Model Coefficients

Feature selection was repeated independently within each of the 25 training partitions. Six features were selected in every partition: V2 S‐wave amplitude, V3 S‐wave amplitude, V1 S‐wave amplitude, V4 R‐wave amplitude and V5 R‐wave amplitude, together with age. The precordial transition zone score during PVC was selected in 23 of 25 partitions and V3 S‐wave width in 22 of 25. Only two further features were selected in any partition—V3 R‐wave width in 4 and aVL R‐wave amplitude in 1—indicating that the retained feature set was highly stable across partitions (Table 2). All selected electrocardiographic features referred to PVC characteristics. Gender was not selected in any partition, nor was PVC QRS duration, nor any sinus rhythm feature.

**TABLE 2:** Features selected and model coefficients.

Feature	Selected (of 25 folds)	*F*‐score	Coefficient
Age (years)	25/25	27.11	+0.100
V2 S‐wave amplitude (PVC)	25/25	23.68	−0.088
V3 S‐wave amplitude (PVC)	25/25	20.11	−0.085
PVC transition zone score	23/25	14.61	−0.077
V1 S‐wave amplitude (PVC)	25/25	16.42	−0.076
V4 R‐wave amplitude (PVC)	25/25	15.42	+0.074
V5 R‐wave amplitude (PVC)	25/25	14.22	+0.073
V3 S‐wave width (PVC)	22/25	12.93	+0.066

*Note:* Logistic regression model coefficients for the eight most important features. Age emerged as the most discriminative feature. The magnitude of each coefficient indicates the feature's relative importance in distinguishing between right and left ventricular outflow tract origin.

In the reference model fitted to the complete cohort, the same eight features were retained. Age was the most discriminative feature (*F*‐score = 27.11), followed by V2 S‐wave amplitude (*F* = 23.68) and V3 S‐wave amplitude (*F* = 20.11). The selected hyperparameters were a regularization strength of 0.01, an L2 penalty and the ‘liblinear’ solver. The corresponding standardized coefficients were: age (+0.100), V2 S‐wave amplitude (−0.088), V3 S‐wave amplitude (−0.085), PVC transition zone score (−0.077), V1 S‐wave amplitude (−0.076), V4 R‐wave amplitude (+0.074), V5 R‐wave amplitude (+0.073) and V3 S‐wave width (+0.066), with an intercept of +0.041 (Table 2). Each coefficient represents the direction and magnitude of a feature's contribution to LVOT versus RVOT classification, with positive values favoring LVOT origin and negative values favoring RVOT origin.

### 3.3 Model Performance

Across the 25 held‐out partitions, the full model, incorporating electrocardiographic features together with age and sex, achieved an accuracy of 0.817 ± 0.143, precision of 0.888 ± 0.122, recall of 0.824 ± 0.206, F1‐score of 0.836 ± 0.139 and ROC‐AUC of 0.917 ± 0.094 (Table 3). Pooling the averaged out‐of‐fold predictions across all 43 patients, the model achieved an accuracy of 0.860 (95% CI 0.744–0.953) and an ROC‐AUC of 0.910 (95% CI 0.800–0.987), with a precision of 0.857, recall of 0.923 and F1‐score of 0.889 (Table 3, Figure 2). Sensitivity for LVOT origin was 0.923 and specificity for RVOT origin was 0.765. The corresponding confusion matrix is presented in Table 4.

**TABLE 3:** Model performance.

Metric	Fold‐wise (mean ± SD)	Patient‐level (95% CI)
Accuracy	0.817 ± 0.143	0.860 (0.744–0.953)
Precision	0.888 ± 0.122	0.857 (0.714–0.968)
Recall	0.824 ± 0.206	0.923 (0.800–1.000)
F1‐score	0.836 ± 0.139	0.889 (0.783–0.966)
ROC‐AUC	0.917 ± 0.094	0.910 (0.800–0.987)

*Note:* Fold‐wise values are the mean ± standard deviation across the 25 held‐out partitions. Patient‐level values derive from the averaged out‐of‐fold predicted probabilities, with 95% confidence intervals by bootstrap resampling (2000 iterations).

**FIGURE 2:**
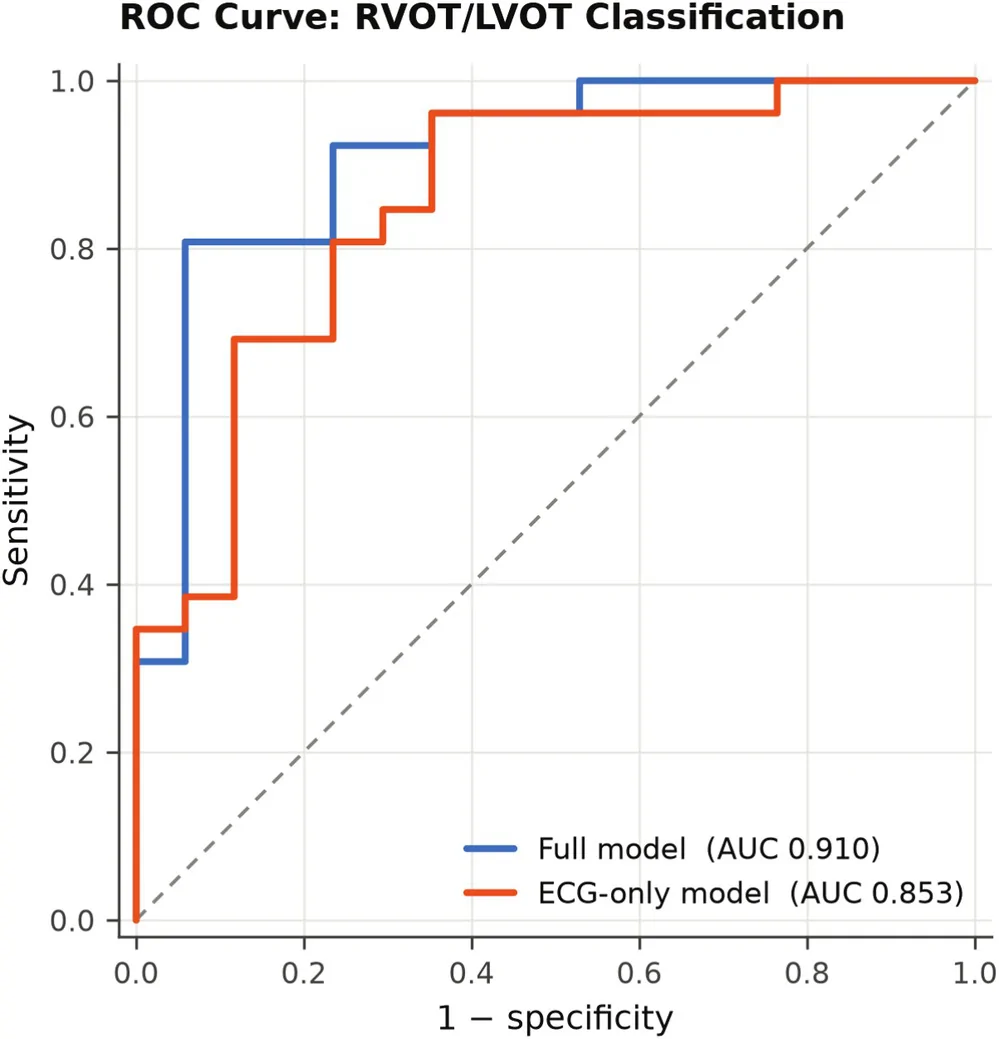
Receiver operating characteristic (ROC) curves for the logistic regression model with corresponding area under the curve (AUC) value.

**TABLE 4:** Confusion matrix, full model.

	Predicted RVOT	Predicted LVOT	Total
Actual RVOT	13	4	17
Actual LVOT	2	24	26
Total	15	28	43

### 3.4 Model Comparison

The ECG‐only model achieved an accuracy of 0.756 ± 0.131 and an ROC‐AUC of 0.876 ± 0.128 across the 25 held‐out partitions, and a pooled accuracy of 0.767 (95% CI 0.628–0.884) and ROC‐AUC of 0.853 (95% CI 0.721–0.958) (Table 3). Compared with the ECG‐only model, the reference model had a ΔAUC of +0.057 (*p* = 0.104). Applied to the same cohort at their originally published cut‐offs, the four conventional algorithms yielded ROC‐AUCs of 0.891 (95% CI 0.779–0.976) for the V2S/V3R index, 0.796 (0.645–0.931) for the R/S‐wave amplitude index, 0.774 (0.614–0.901) for the transitional zone index and 0.704 (0.532–0.866) for the V2 transition ratio (Table 5). Compared with the reference model, ΔAUC was +0.018 (*p* = 0.609) for the V2S/V3R index, +0.112 (*p* = 0.075) for the R/S‐wave amplitude index, +0.130 (*p* = 0.111) for the transitional zone index and + 0.190 (*p* = 0.033) for the V2 transition ratio.

**TABLE 5:** Comparison with published ECG algorithms.

	AUC (95% CI)	ΔAUC vs. reference model	*p*
Reference model	0.910 (0.800–0.987)	—	—
ECG model	0.853 (0.721–0.958)	0.057	0.104
V2S/V3R index	0.891 (0.779–0.976)	0.018	0.609
R/S amplitude index	0.796 (0.645–0.931)	0.112	0.075
Transitional zone index	0.774 (0.614–0.901)	0.130	0.111
V2 transition ratio	0.704 (0.532–0.866)	0.206	0.033

*Note:* Published algorithms applied at their originally reported cut‐offs; as they involve no fitting, cross‐validation does not apply and they were evaluated in the whole cohort. Differences in AUC assessed by DeLong's test for correlated ROC curves.

## 4 Discussion

In this study, we developed a machine learning model incorporating electrocardiographic and demographic features to differentiate between LVOT‐ and RVOT‐originating PVCs. Feature selection, repeated within each training partition, identified a stable set of eight predictors, of which age was the most discriminative and was retained in every partition. Increasing V1, V2, and V3 S‐wave amplitudes during the PVC were associated with RVOT origin, as was a later precordial transition zone during PVC, whereas higher V4 and V5 R‐wave amplitudes favored LVOT origin. Across the 25 held‐out partitions, the model achieved an accuracy of 0.817 ± 0.143 and an ROC‐AUC of 0.917 ± 0.094; using the pooled out‐of‐fold predictions, accuracy was 0.860 (95% CI 0.744–0.953) and ROC‐AUC 0.910 (95% CI 0.800–0.987).

When compared with previously published electrocardiographic algorithms applied to the same cohort, the model achieved higher discrimination than all four, although this difference reached statistical significance only against the V2 transition ratio (ΔAUC +0.190, *p* = 0.033). Notably, the V2S/V3R index performed comparably to the model (AUC 0.891 versus 0.910; ΔAUC +0.018, *p* = 0.609), indicating that a single, readily measured ratio captures much of the discriminative information available in this cohort. The model restricted to electrocardiographic features alone did not exceed this index (ΔAUC −0.038, *p* = 0.441), and the addition of age improved discrimination without reaching statistical significance (ΔAUC +0.057, *p* = 0.104). These findings suggest that the principal contribution of a machine learning approach in a cohort of this size lies in the systematic integration of demographic and electrocardiographic information rather than in a demonstrable gain over the best existing index.

To the best of our knowledge, this is the first model that quantifies and directly compares the relative contributions of demographic variables and electrocardiographic characteristics in differentiating LVOT‐ from RVOT‐originating PVCs. Our results are in agreement with a deep learning model described by Nakasone et al., which demonstrated that PVC‐related features contribute more strongly than sinus rhythm features to arrhythmia site‐of‐origin differentiation (Nakasone et al. 2024); in the present analysis no sinus rhythm feature was selected in any of the 25 training partitions. In that study, features derived from leads V2 and V3 were predominantly associated with right‐sided origin, whereas features from leads V4 and V5 showed stronger contributions to left‐sided origin, and precordial lead–derived features contributed more prominently than limb lead–derived features—a pattern reproduced by the coefficient directions observed here. In accordance with these findings, a previously published convolutional neural network–based model demonstrated that the relative contribution to site‐of‐origin prediction was higher than average for leads V1 and V2 in cases of RVOT‐originating arrhythmias (Nakasone et al. 2022), and Zheng et al. demonstrated that voltage values in lead V1 were the most important variable for separating arrhythmia origin (Zheng et al. 2022).

Further, our results reinforce the concept that demographic characteristics can be reliably utilized to help differentiate the site of origin of PVCs. Age was the single strongest discriminator in this cohort, with a median of 41.0 years in RVOT‐ and 67.8 years in LVOT‐originating arrhythmias (*p* < 0.001) and was selected in every training partition. Penela et al. previously proposed a hybrid score that integrates demographic parameters—including comorbidities—with electrocardiographic features, demonstrating superior diagnostic performance compared with traditional algorithms based solely on ECG criteria (Penela et al. 2023). Tanaka et al. reported that patients with LVOT‐originating PVCs are generally older than those with RVOT‐originating PVCs (Tanaka et al. 2011). This hybrid approach addresses several limitations arising from the exclusive use of ECG features, including the close anatomical proximity of the ventricular outflow tracts and the presence of myocardial fiber connections between them, which often result in overlapping electrocardiographic patterns. It also diminishes the influence of cardiac rotation, lead position, preferential conduction and interobserver variability in ECG measurements.

Overall, our results support the anatomical rationale that right‐sided ECG leads are more informative for RVOT arrhythmia detection, while also illustrating that careful feature selection can reduce dimensionality without sacrificing discriminative power. Reporting the frequency with which each feature was selected across partitions provides an explicit measure of the stability of that selection, which single‐split analyses cannot offer. This interpretable approach complements prior deep learning and ECG‐based studies by providing clinically meaningful insight into the relative importance of individual features.

This study has several limitations, most notably the small sample size. Eight features were retained for 17 patients in the minority class, and although all preprocessing, feature selection and hyperparameter optimization were nested within the cross‐validation procedure, confidence intervals remained wide and only one of the five pairwise comparisons performed reached statistical significance. The present findings should therefore be regarded as hypothesis‐generating. In addition, the retrospective design could limit the completeness and accuracy of information regarding arrhythmia site of origin and periprocedural characteristics. Moreover, in the presence of an intramural arrhythmogenic focus, ablation is often performed at the site of preferential conduction rather than at the true site of origin; consequently, the ablation target may not accurately reflect the actual arrhythmogenic source.

## 5 Conclusions

This study demonstrates that a machine learning model integrating demographic and electrocardiographic features showed good discrimination between RVOT‐ and LVOT‐originating PVCs, with age emerging as the most stable and discriminative predictor. Precordial ECG features, particularly from leads V1–V5, were identified as highly discriminative. The model achieved numerically higher discriminative performance compared to the ECG‐only model and the traditional discriminative algorithms. These findings highlight the potential of interpretable, feature‐based machine learning models to support non‐invasive arrhythmia localization in clinical practice. External validation on independent cohorts is needed to confirm generalizability.

## Author Contributions

Conceptualization: Michail Botis, Dimitris Tsiachris. Data curation: George G. Botis, Ioannis Doundoulakis. Methodology: Athanasios Kordalis, Christos‐Konstantinos Antoniou. Writing – original draft: Aikaterini‐Eleftheria Karanikola, Lamprini Iro Bartsioka. Writing – review and editing: Christos‐Konstantinos Antoniou. Visualization and tables: Aristomenis Koumpanakis. Supervision: George K. Matsopoulos, Konstantinos Tsioufis. Validation and Final Approval: All authors.

## Funding

The authors have nothing to report.

## Conflicts of Interest

The authors declare no conflicts of interest.

## Data Availability

The data that support the findings of this study are available from the corresponding author upon reasonable request.
